# The role of exploitation in the establishment of mutualistic microbial symbioses

**DOI:** 10.1093/femsle/fnz148

**Published:** 2019-07-04

**Authors:** Megan E S Sørensen, Chris D Lowe, Ewan J A Minter, A Jamie Wood, Duncan D Cameron, Michael A Brockhurst

**Affiliations:** 1 Department of Animal and Plant Sciences, University of Sheffield, Sheffield S10 2TN, UK; 2 Centre for Ecology and Conservation, University of Exeter, Penryn Campus, Cornwall TR10 9FE, UK; 3 Department of Biology, University of York, York YO10 5DD, UK; 4 Department of Mathematics, University of York, York YO10 5DD, UK

**Keywords:** microbiology, experimental evolution, microbial symbioses

## Abstract

Evolutionary theory suggests that the conditions required for the establishment of mutualistic symbioses through mutualism alone are highly restrictive, often requiring the evolution of complex stabilising mechanisms. Exploitation, whereby initially the host benefits at the expense of its symbiotic partner and mutual benefits evolve subsequently through trade-offs, offers an arguably simpler route to the establishment of mutualistic symbiosis. In this review, we discuss the theoretical and experimental evidence supporting a role for host exploitation in the establishment and evolution of mutualistic microbial symbioses, including data from both extant and experimentally evolved symbioses. We conclude that exploitation rather than mutualism may often explain the origin of mutualistic microbial symbioses.

## Teaser

The authors review the theoretical and experimental evidence supporting exploitation as an alternative route to the evolution of mutualistic symbioses.

## INTRODUCTION

Symbiosis – ‘the living together of unlike organisms’(De Bary 1879) – encompasses a broad range of species interactions, including both parasitism (+/– fitness interactions) and mutualism (+/+ fitness interactions). Whilst the evolutionary rationale for parasitism is straightforwardly explained by the self-interest of the parasitic partner, explaining the origin of mutualistic symbiosis is more challenging. The immediate fitness gains of cheating are expected to outweigh the potential long-term fitness benefits of cooperation, producing a ‘tragedy of the commons’ (Hardin 1968; Rankin, Bargum and Kokko 2007). Therefore, both in long-established associations and in the establishment of new relationships, evolutionary conflict and breakdown of mutualistic symbiosis is ever likely, since each partner is under selection to minimise its investment in the integrated symbiotic unit (Perez and Weis 2006; Sachs and Simms 2006). Nevertheless, mutualistic symbiotic relationships are abundant, taxonomically widespread, ecologically important in a wide range of habitats, economically important in agricultural systems and, consequently, underpin the biodiversity and function of both natural and man-made ecosystems (Bronstein 2015; Powell and Rillig 2018).

Mutualistic symbiosis can accelerate evolutionary innovation through the merger of once independent lineages, providing species with new ecological traits and allowing them to inhabit previously inaccessible ecological niches (Wernegreen 2004; Kiers and West 2015). A classic example of this is nutrient trading, where the partners exchange compounds that are otherwise difficult or impossible for them to acquire. These include aphids with their obligate endosymbiont *Buchnera aphidicola* that exchange essential amino acids (Moran *et al*. 2003), and land plants with arbuscular mycorrhizal fungi where fixed carbon is exchanged for phosphate and organic nitrogen (Pfeffer *et al*. 1999). Besides exchanging nutrients, mutualistic symbioses can involve a wide range of benefits, including the production of antibiotics (Currie *et al*. 1999), luminescence (Tebo, Scott Linthicum and Nealson 1979), photoprotection (Hörtnagl and Sommaruga 2007) and protection from predation (Tsuchida *et al*. 2010). Since many of these potential benefits may only be required in particular environments or at particular times, many symbioses vary ecologically across a continuum from mutualism to parasitism (Heath and Tiffin 2007; Wendling, Fabritzek and Wegner 2017). Indeed, some organisms may only engage in symbiosis when in nutrient-deficient environments (Muscatine and Porter 1977; Johnson 2011).

Mutualistic symbiosis involves a shift in individuality as two unrelated species evolve inter-dependence and transition to function as a single organism (Szathmáry and Smith 1995; Estrela, Kerr and Morris 2016). In nature, the degree of dependence varies extensively both within and between symbioses (Minter *et al*. 2018). Dependence can range from obligate associations with mutually dependent partners, through asymmetrically dependent associations where only one species is unable to survive alone, to fully facultative associations where both species can survive alone. Comparative studies suggest that mutual dependence is more likely to evolve in vertically inherited symbioses, where the fitness interests of both species become aligned (Fisher et al., 2017). Transitions in individuality are, however, fraught with evolutionary conflict, and the merger of two independent organisms is rarely seamless and never selfless. Conflict is likely to be greatest during the establishment of new symbioses, before the partners have been able to evolve complex mechanisms required to align their fitness interests.

Explaining the establishment of mutualistic symbioses is therefore challenging, and this is the focus of our review. As we shall explain in the subsequent section, the conditions for mutualistic symbioses to establish through mutualism alone are highly restrictive, and thus several alternative mechanisms have been proposed (Garcia and Gerardo 2014; Keeling and McCutcheon 2017). One of these is that mutualistic symbioses evolve from parasitisms. This transition can occur in two directions. First, the smaller parasitic partner living in or on the larger host can evolve reduced virulence to eventually become beneficial to its host (King *et al*. 2016; Shapiro and Turner 2018; Tso *et al*. 2018). Sach *et al*. (2011) used phylogenetic reconstruction to predict whether bacterial symbionts originated as mutualists or parasites. For 42 beneficial bacterial symbionts, they inferred that 32 had originated as parasitic whilst only 9 had originated as mutualists (with 1 case remaining ambiguous), suggesting that parasitism is a more common route than mutualism to mutualistic symbiosis. Second, the larger host partner could capture and exploit the smaller beneficial partner, which would otherwise grow faster outside of symbiosis. This is a special case of parasitism known as host exploitation, which has been far less well-studied. In this review, we gather together the evidence supporting a role for host exploitation in the establishment of mutualistic microbial symbiosis.

## THEORETICAL STUDIES OF SYMBIOSIS: MUTUALISM VERSUS EXPLOITATION

### The paradox of mutualism

Mutualisms are abundant throughout the tree of life despite their inherent evolutionary conflicts, and this disparity is considered the paradox of mutualism. The paradox of mutualism has been well explored using theoretical models that aim to discover the evolutionary stable strategies of mutualistic symbiosis. The reciprocal exchange of services/goods within mutualisms make them a specific form of group cooperation. There are two primary evolutionary explanations for group cooperation. Within a species, kin selection explains that helping related individuals provides inclusive fitness benefits to the actor (following Hamilton's rule (Hamilton 1964)). Alternately for non-relatives, game theory has provided the strategic alliance model, which is based around reciprocity and includes the Tit-for-Tat strategy (Axelrod 1984). Frank (1996), however, highlighted that the evolution of interspecific symbiosis cannot be explained by either of these models; kin selection is not applicable because the interaction is between unrelated individuals from different species, and the strategic alliance model fails because it requires memory of past interactions, the recognition of individuals and is dissipated by forms of mixing. The traditional explanations for cooperation are, therefore, insufficient to explain the evolutionary stability of symbioses.

Theoretical work has consequently focused on mutualism-specific explanations, and a key process underlying much of this work is finding mechanisms that align the partners’ fitness interests. Herre *et al*. (1999) proposed that this alignment could be achieved by ‘conflict avoidance factors’, which include vertical transmission, genetic uniformity of symbionts, population spatial structure and obstructions to alternative free-living states. The influence of these factors has been explored by theoretical models, particularly vertical transmission that aligns the reproductive interests of the partners (Yamaura (1993)). For reproductive interests to be fully aligned, both absolute co-dispersal and reproductive synchrony are required as part of vertical transmission (Frank 1997). If achieved, this reduces within-host competition between symbionts and stabilises the mutualism because the reproductive success of the symbiont is perfectly aligned to that of its host. Vertical inheritance is common in well-established, obligate symbiotic partnerships and is associated with greater dependence (Fisher *et al*. 2017). It is not, however, ubiquitous and there are many stable mutualisms that maintain horizontal transmission. For example, *Vibrio fischeri* and bobtail squids (Visick and Ruby 2006), Rhizobia and legumes (Sprent, Sutherland and Faria 1987), and *Endoriftia persephone* and tube worms (Nussbaumer, Fisher and Bright 2006). Consequently, it is clear that while conflict avoidance factors help to promote stability of some interactions, they are neither necessary nor sufficient for the evolutionary stability of mutualistic symbioses (Genkai-Kato and Yamamura 1999).

Frank (1995) provided a solution to the paradox of mutualism by developing a model centred on policing strategies, which repressed competition and reduced the benefits of cheating to ensure the fair distribution of resources. Furthermore, the results of the extended policing model (Frank 1996) showed that variation in individual resources altered the degree of investment in policing, with well-supplied individuals doubling their policing investment and poorly supplied individuals not investing at all. The theoretical prediction for the role of policing in maintaining mutualistic symbioses has been supported by numerous occurrences in a wide-range of natural systems. For example, partner sanctions in the legume–rhizobium symbiosis (Kiers *et al*. 2003), partner choice in the yucca–yucca moth symbiosis (Bull and Rice 1991), partner fidelity in solitary wasp–Streptomyces symbiosis (Kaltenpoth *et al*. 2014) and screening in the bobtail squid–*Vibrio fischeri* symbiosis (McFall-Ngai and Ruby 1991; Archetti *et al*. 2011).

Following Frank's first policing models, there has been extensive development of theory exploring the evolution of mutualism. The current consensus is that stabilising mechanisms, such as the various policing strategies, vertical transmission and other conflict avoidance factors, provide solutions to the paradox of mutualism (for extensive reviews of the topic, see Sachs *et al*. (2004); Leigh (2010) and Archetti *et al*. (2011)). However, while it is clear that these complex adaptations play a crucial role in the maintenance of extant mutualistic symbioses, it is unlikely that they can explain the origin of new symbioses because here there is little time for such complex stabilising mechanisms to evolve. The pre-existence of such traits, allowing for their co-option for the purpose of stabilising symbiosis, may be a pre-requisite for the establishment of symbiosis. For instance, one can imagine that partner-choice could evolve from pre-existing feedback mechanisms and may even provide the selective environment from which the symbiosis establishes (Frederickson 2013). However, given that complex stabilising mechanisms are not ubiquitous this seems unlikely to be a general explanation. Moreover, elaborate host–symbiont interactions, such as the bobtail squid–*Vibrio fisheri* multistage screening process, must have evolved subsequent to establishment, even if the fundamental aspects were pre-adaptations. It is more parsimonious therefore to assume that important limitations exist as to the conditions where mutualism can act as an establishment mechanism for mutualistic symbiosis.

### Exploitation as an alternative route to symbiosis

An alternative route to the establishment of mutualistic symbiosis was proposed by Law and Dieckmann (1998). This model predicted that exploitative relationships wherein a host exploits a ‘victim’ species which it acquires by horizontal transmission can evolve into stable mutualistic symbioses with vertical transmission simply through natural selection to increase individual fitness. The key requirement for this outcome was that the free-living victim pays a cost to defend itself from being captured by the host. In this scenario, there is a trade-off for the victim, who either uses resources to defend itself or to provision the exploitative host. Depending on the relative magnitude of these trade-offs, it is possible that the victim has higher fitness in symbiosis. In this case, the evolution of vertical transmission is advantageous to both partners as the victim has a higher reproductive rate in symbiosis than when free-living, where it must pay a high cost of defence. However, it remains the case that the victim's optimal state would be to be free-living with no interaction with the exploiter and thus paying neither of these costs. The model demonstrated that if the trade-off is sufficiently strong, the evolution of stable symbiosis can be advantageous to both partners even in an exploitative relationship. Furthermore, once vertical transmission has evolved it becomes much harder for the victim to escape the host, and the victim can become trapped in the symbiotic state. It is important to note that this interaction has now become a mutualistic symbiosis; the victim provisions the host to the host's benefit, whilst the victim's reproductive rate in symbiosis now exceeds that which is achievable in free-living environments containing the host.

Because host exploitation does not require symmetric mutual benefits at the outset nor complex stabilising mechanisms to allow establishment, it offers a simpler explanation for the emergence of mutualistic symbiosis. Once mutualistic symbiosis is established, further stabilising mechanisms could evolve to prevent its breakdown. Thus mutualism-stabilising mechanisms may often be a secondary phenomenon, arising to further enforce originally exploitative but now mutualistic symbioses.

## EXPLOITATION IN ACTION

Empirical data on the establishment of mutualistic symbioses are rare because studying this process experimentally is challenging. The extant mutualistic symbioses we observe in nature are the products of co-evolution and no longer in the establishment phase. Furthermore, for obligate mutualistic symbioses it may be impossible to separate the partners and therefore untangle the costs/benefits that each of the symbiotic partners derive. Nonetheless, there are several mutualistic microbial symbioses that are amenable to experimental study, and two main experimental approaches. The first approach is to study extant facultative associations that remain experimentally tractable and allow the direct measurement of the relative costs and benefits of both the free-living and symbiotic states. The second approach is to experimentally evolve newly formed symbioses in the laboratory to explore the environmental conditions that promote their establishment and stability (Hoang, Morran and Gerardo 2016). We review the data from both approaches in the following section.

### Experiments with extant facultative mutualistic microbial symbioses

**Figure 1.**
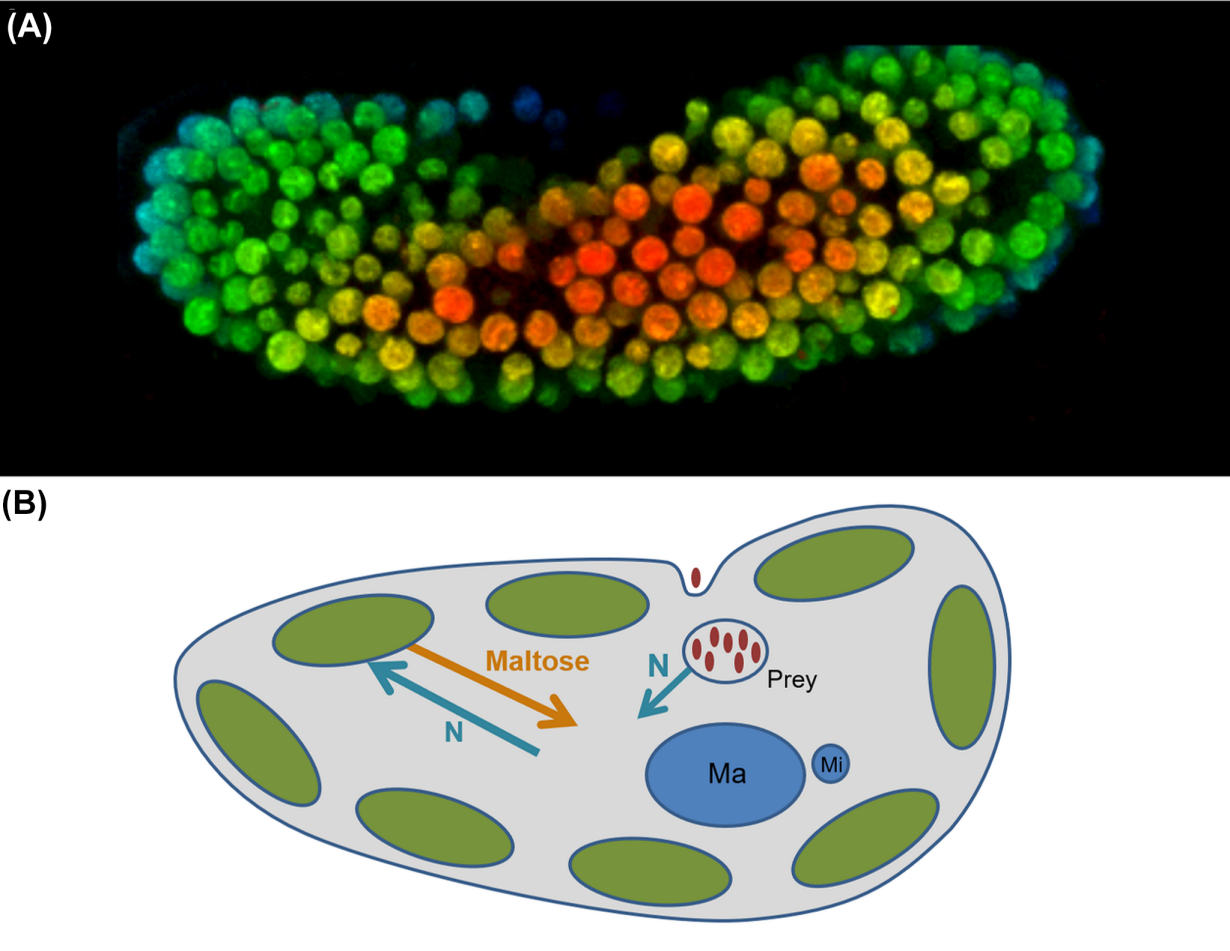
*Paramecium bursaria* and *Chlorella* endosymbiosis. A. Z-stack of confocal sections of the chlorophyll autofluorescence of *Chlorella* endosymbionts within one *Paramecium bursaria* cell. With colour representing the intensity of fluorescence and therefore the position of the *Chlorella* in the Z-plane. B. Diagram of the relationship, showing the nutrient exchange with the transfer of maltose from the *Chlorella* in exchange for organic nitrogen (denoted as ‘N’ as the identity of this compound is currently unknown). Ma = macronucleus; Mi = micronucleus.

One of the best studied facultative mutualistic microbial symbioses is that between the single-celled ciliate host *Paramecium bursaria* and its green alga symbiont, *Chlorella*. This classical photosymbiosis is founded upon the exchange of fixed carbon from the photosynthetic algae in return for organic nitrogen from the host (Fig. 1). It has been estimated that the *Chlorella* endosymbionts release 57% of their fixed carbon to the host (Johnson 2011), primarily as maltose (Ziesenisz, Reisser and Wiessner 1981). The nitrogen source is not yet verified; current candidates include amino acids (Kato, Ueno and Imamura 2006; Kato and Imamura 2008b), nucleic acid derivatives (Soldo, Godoy and Larin 1978; Shah and Syrett 1984) and ammonia (Albers, Reisser and Wiessner 1982).

Crucially, while the symbionts are inherited vertically with tight cell cycle synchrony, the partners can be separated by sonication/chemical treatment (Kodama and Fujishima 2008, 2011, 2012) allowing the costs and benefits of symbiosis versus free-living to be directly compared. For hosts, the benefit of symbiosis increases with light intensity, such that while it is costly to harbour symbiotic algae in the dark (i.e. symbiont-free hosts grow faster than symbiotic hosts), these costs are outweighed at higher light intensity such that symbiosis is highly beneficial for hosts in high light. In contrast, symbiosis is never beneficial for the alga: free-living algal growth rates increase monotonically with light intensity and at all light levels exceed those of symbiotic algae. Moreover, hosts impose tight control on algal symbiont load (i.e. the number of algal symbionts per host cell) which peaks at low light, and is reduced both in the dark and at high light intensity (Lowe *et al*. 2016). A mathematical model of the symbiosis showed that hosts manipulate symbiont load in this way to maximise their return from nutrient trading, effectively minimising their nitrogen cost for each molecule of carbon they gain from their algal symbionts (Dean *et al*. 2016). Indeed, measurements of algal photosynthetic efficiency suggested that algal symbionts were more nitrogen-starved than their free-living counterparts (Lowe *et al*. 2016). Similar patterns of cost:benefit and host control were observed across a range of geographically diverse isolates (Minter *et al*. 2018).

The mechanism of the control in this relationship is likely to be multifaceted, but in large part is thought to be due to host digestion. Host selection in the establishment of the symbiosis specifies which *Chlorella* are packaged into vacuoles and re-located, while all others are digested (Kodama and Fujishima 2011, 2014). Even once established, complete darkness or chemical inhibitors, both of which prevent *Chlorella* photosynthesis and therefore stop the carbon supply to the host, lead to the eventual loss of *Chlorella* symbionts, through either digestion or egestion (Karakashian 1963; Kodama and Fujishima 2008). In addition, cell division of symbiotic *Chlorella* is tightly regulated and has been linked to host cytoplasmic streaming (Takahashi *et al*. 2007). Furthermore, metabolic processes are believed to actively influence the exchange process, for instance host Ca^2+^ inhibits serine uptake into *Chlorella* and glucose increases the uptake (Kato and Imamura 2008a, 2008b). If the symbiont's maltose is broken down to glucose by the host, then this control process would facilitate a reward system for more co-operative symbionts. The multiple control processes identified to date are all host-derived, supporting the idea that this symbiosis was founded upon exploitation.

Phylogenetic analysis shows that symbiotic and free-living *Chlorella* form polyphyletic groups (Hoshina and Imamura 2008; Summerer, Sonntag and Sommaruga 2008), indicating multiple transitions to and from symbiosis. Moreover, diverse isolates of *P. bursaria–Chlorella* vary in their degree of dependence; from completely facultative associations to obligate mutual dependence, via asymmetric dependence where hosts depend on symbionts but not vice versa (Minter *et al*. 2018). Taken together, these experimental data suggest that the nutrient trading relationship between the ciliate and the alga is exploitative rather than mutualistic, benefiting the host (Lowe *et al*. 2016). Additional selective forces may be required therefore to explain the benefit of symbiosis for the alga, and while several have been proposed, including photoprotection and escape from viral predation (Reisser *et al*. 1991; Summerer *et al*. 2009; Esteban, Fenchel and Finlay 2010), this interaction proves that a stable, even sometimes obligate, symbiosis can evolve from exploitation.

Other similar symbioses also appear to be founded upon exploitation. For example, for scleractinian corals and the dinoflagellate algae *Symbiodinium* there is evidence of asymmetry in the fitness effects of symbiosis upon the partners. The algal growth rate is reduced from a free-living doubling time of 3 days to a symbiotic doubling time of between 70 and 100 days (Wilkerson, Kobayashi and Muscatine 1988). Whereas hosts experience increased growth rates in symbiosis. Further support for the idea that this association is exploitative is provided by the asymmetry of the nutrient exchange: whilst the algal symbiont provides ∼95% of its photosynthate to the host, in return they are kept in a nitrogen-starved state by the host (Smith and Muscatine 1999; Dubinsky and Berman-Frank 2001). Similarly, studies on lichen symbioses and the partnership between chemosynthetic bacteria and their invertebrate hosts have also reported reduced symbiont growth rates in symbiosis compared to free-living (Ahmadjian 1993; Combes 2005). Additionally, the association of Acantharia marine protists with haptophyte algae is also believed to be a form of farming, whereby only the host benefits (Decelle 2013). What these interactions have in common is that they feature a producer living within a consumer. In both the coral and *P. bursaria* symbioses, the algal symbionts are ‘engulfed’ during establishment and therefore do not actively enter symbiosis. In symbiosis, the algae are contained within a host membrane, enabling the host to control provisioning of resources. This inequality of control may be a defining feature of apparently mutualistic symbioses founded upon exploitation.

### Experimental evolution of microbial symbioses

Experimental evolution provides an unparalleled window into evolutionary processes by allowing their observation in real time from defined genetic and phenotypic starting points under controlled conditions in the laboratory. While simplified lab environments preclude direct comparisons to nature, they allow key variables to be separated from the myriad of confounding variables in the field, providing a way to unambiguously separate the proximate and ultimate causes of symbiosis (Mazancourt, Loreau and Dieckmann 2005).

To date there are only few examples of experimentally evolved establishments of novel symbiotic relationships. Jeon (1972) reported the first instance of an intracellular obligate parasite evolving to become a mutualistic symbiont. The experiment used *Amoeba discoides* that had become spontaneously infected with rod-shaped bacteria and these were then cultured together, without any selection for symbiosis, for five years. At first, the bacteria were harmful; the infected amoebae grew slower, were more sensitive to starvation, were smaller and some hosts cells were killed upon infection. However, after five years, the infected amoebae grew normally despite carrying the same number of bacteria cells. Crucially, this was not due simply to the evolution of reduced virulence by the bacterium. Nuclear transfer experiments swapped the evolved nucleus and cytoplasm with that of the ancestor and demonstrated that the evolved nucleus could now not survive without the coevolved bacterial symbiont. Thus, a mutualistic and obligate symbiosis had evolved from a parasitism.

More recently, Nakajima et al. (2009, 2015) established long-term microcosms containing a green alga (*Micractinium* sp., formally *Chlorella vulgaris)*, a bacterium (*Escherichia coli*), and a ciliate (*Tetrahymena thermophila)*. The experiment was maintained without external addition of resources and without transfer to fresh medium for over five years and therefore formed a self-sustaining ecosystem. Over the course of the experiment the free-living algae diversified into two distinct forms. One of these was a non-aggregating type that formed an endosymbiotic association with *Tetrahymena* as its host, whereas an aggregate forming type lived outside of *Tetrahymena* cells but formed a symbiotic association with the *E. coli*. The algal aggregation phenotype was negatively correlated with *Tetrahymena* longevity in coculture, suggesting that only non-aggregating algae improved host fitness. Potentially underpinning this host benefit, the evolved endosymbiotic algae excreted more glycerol and sucrose, and contained more photopigments than the ancestral clone (Germond *et al*. 2013). The evolved free-living algae adapted to the free-living environment and outcompeted any endosymbiotic algae that escaped symbiosis. This suggests that a trade-off between adaptation to the free-living versus the symbiotic environment may frequently enforce interspecific cooperation and thus stabilise symbiosis, and is conceptually similar to the trade-off proposed by Law and Dieckmann (1998).

Although additional experimental evolution studies are clearly needed, it is intriguing that both studies to date support the role for exploitation in the establishment of symbioses that evolve become mutualistic. Both experiments suggest a key role for trade-offs between symbiotic and free-living environments in driving the emergence of mutualistic symbiosis, as predicted by Law and Dieckmann (1998). These experiments were essentially observational in design, lacking treatments to compare the effects of environmental variables. Experiments manipulating key environmental parameters likely to affect symbiosis, such as the potential for horizontal transmission or the free-living mortality rate, will be an important next step towards understanding the environmental drivers of the establishment of symbiosis.

## CONCLUSION

Both the theoretical and empirical evidence support the role for parasitism or exploitation in the establishment of symbioses, and the later evolution of mutual benefit. Establishment through exploitation provides a simple explanation for the establishment of symbiosis because it does not require complex stabilising mechanisms to repress conflict. Exploitation may be especially prevalent among associations where the smaller partner is engulfed by a larger host and enclosed in the host membrane. In such associations, it is clear from the available experimental data that the core nutrient exchange between partners does not in itself provide mutual benefits. It is likely that fitness trade-offs between the symbiotic and free-living environments play a key role in enforcing exploitative symbioses, and may lead to the eventual emergence of dependence and mutual benefit through the loss of fitness in the free-living state.
